# Metaphors of War in Effective and Ineffective Coping of Medical Directors of COVID-19 Wards in Public Hospitals

**DOI:** 10.3389/fpubh.2022.830266

**Published:** 2022-04-14

**Authors:** Lior Naamati-Schneider, Gillie Gabay

**Affiliations:** ^1^Department of Health Systems Management, Hadassah Academic College Jerusalem, Jerusalem, Israel; ^2^Department of Interdisciplinary Studies, Achva Academic College, Arugot, Israel

**Keywords:** COVID-19, coping, clinicians, discourse analysis, hospitals, mapping, social constructivism, war metaphors

## Abstract

The COVID-19 pandemic has challenged medical professionals worldwide with an unprecedented need to provide care under conditions of complexity, uncertainty, and danger. These conditions, coupled with the unrelenting stress of overwhelming workloads, exhaustion, and decision-making fatigue, have forced clinicians to generate coping mechanisms. This qualitative study explored the use of metaphors as a coping mechanism by clinical directors of COVID-19 wards in Israeli public general hospitals while they were exposed to death and trauma throughout the pandemic's first wave in Israel (March to June 2020). The study employs discourse methodology and metaphor mapping analysis to capture the personal, organizational, and social dimensions of effective and ineffective processes of coping with an extreme health crisis. Analysis revealed that the metaphors that clinical directors used reflect a dual process of mediating and generating the social construction of meaning and facilitating effective and ineffective coping. Effective coping was facilitated by war metaphors that created a sense of mission and meaningfulness at both the organizational and the individual levels. War metaphors that generated a sense of isolation and sacrifice intensified helplessness and fear, which undermined coping. We propose actionable recommendations to enhance effective coping for individuals and organizations in this ongoing pandemic.

**“*When the cannons and the corona roar, the muses are silent, and the doctors on the frontline battle against an invisible enemy, in a fog of uncertainty.”***

## Background

Health systems and medical staff function under extreme, constantly changing conditions (1). When the COVID-19 pandemic began, there was no treatment protocol or understanding of the medical ramifications. These factors, combined with overwork, fatigue, and intensive exposure to death and trauma, heightened the challenges of working under pressure with staff shortages and insufficient medical equipment (1–3). Doctors and medical teams struggled to cope (4).

While the study of the linguistic, therapeutic, and cognitive mechanisms used by patients to cope with health crises is well-developed, research on the staff's use of linguistic mechanisms for coping with crises is scant (5–7). The current study aims to help fill this gap by examining the use of metaphors and framing of the situation as coping mechanisms used by directors of COVID-19 wards in public hospitals in Israel, upon the outbreak of the pandemic in Israel and throughout its first wave (March–June 2020). This study highlights the effectiveness and ineffectiveness of metaphors in coping with the crises and traumas to which doctors and medical staff were exposed. Understanding the role of metaphors in effective coping may enable the development of intervention programs within the organization. Such staff education may enhance medical teams' coping with stress in both routine work and in crises and may help them build effective coping mechanisms.

### The Theoretical Anchor

Conceptual metaphor theory (CMT) is the theoretical framework of this study (8, 9). This theory views metaphors as both a stylistic linguistic phenomenon that describes how we speak and think about one thing in terms of another (10) and as cognitive behavioral phenomena (cognitive arrays) influenced by the individual's socio-cultural experiences. According to CMT (8, 9), metaphors are cognitive structures that conceptualize complex thought by using familiar content worlds drawn from everyday experiences: Concepts from one area (the target area) that the speaker is trying to conceptualize are represented by concepts from an area that is familiar to the speaker from everyday mental and physical experience (the source area). Thus, source areas that are created by everyday experiences serve to conceptualize the target area, which is abstract, difficult, loaded, and unfamiliar (11). An example is the common metaphor *Life is a journey*. The target area is *life*, and the source area that describes and clarifies the target area is *a journey* (12).

Metaphors are generally based on universal experience, but in many senses, they are also culture- and context-dependent. The choice of the source area and its correspondence to the target area are linked to cultural differences between groups (12). Metaphors are a shared cognitive cultural resource and are determined by local culture, discourse, and social elements combined with individual experiences (11–13). Therefore, the analysis of metaphors cannot be separated from their social environment (12).

Metaphors influence cognitive processes and action, in a bidirectional manner. That is, conceptual metaphors and figurative language influence each other in a process that not only reflects thought but also generates thought and action (14). Thus, language is key in social interaction, mediates bidirectionally between recognition and awareness, and frames reality while generating meaning (14, 15). The framing of reality leads to a different view of a given situation and influences human behavior. For example, framing an illness as a war or a journey will shape individuals' view of their illness and their behavior (16).

Conceptual metaphor theory emphasizes metaphors as a shared cognitive resource that shapes cognition and is therefore used frequently in discourse. Combining CMT with the study of metaphors in discourse makes it possible to identify metaphoric structures in discourse and examine their context for those who have the shared metaphorical cognitive-cultural resource that reflects combinations of cognition, feeling, society, and culture. The researcher's work is to recognize the source areas through the discourse as they represent the dynamic use of the language in a given context (17–21).

### The Use of Metaphors in Medical Contexts

Health providers, doctors, and medical staff often use metaphors to illustrate medical topics to patients (22, 23). Using metaphors mediates difficult experiences of uncertainty, anxiety, and fear of death. Doctors' use of metaphors has been linked to better relations between doctors and patients (22). Patients diagnosed with various types of cancer have bridged the gaps in understanding and communication by means of metaphors (24), and using metaphors has enabled patients to create order and logic in their world, which had suddenly become chaotic (22, 25). Militaristic metaphors are widely used in the field of medicine by both medical staff and patients (5, 7, 26, 27). War-related expressions such as “attacking” and “the enemy” are common (28). The use of violent military metaphors has occasionally impeded healing by generating antagonism toward the perception of the doctor and of medicine as life-saving (25, 29–31).

Because conceptual metaphors are created and exist on the basis of environment- and culture-dependent individual experiences (12), an analysis of metaphors in the coping processes of ward directors in Israel requires a description of their cultural context.

### Israeli Society: The Cultural Background

The Israel Defense Forces are part of the life experience of Israel's citizens, including doctors and medical staff. Military service is compulsory and constitutes a milestone for citizens. Therefore, militarism, military language, and military expressions and metaphors seep into the language, thought, and behavior (32, 33). Studies show the existence of three types of militarism in Israeli society: the aggressive, the cultural, and the cognitive (32). These dimensions can exist separately or in various combinations, so that militarism penetrates to the state of mind both structurally and culturally. Intensive involvement in military action provides a unique natural arena for a thriving war discourse: Normalizing war and militaristic concepts underlies the Israeli discourse (34, 35).

This militarism, which is part and parcel of the Israeli mentality, provides survival as a justification in relation to many topics discussed in Israeli society (36), for example, self-sacrifice, sanctification of death, and the encouragement of childbirth (37). During the COVID-19 period, such language appeared in the media, where it was used to justify and intensify the collection of medical data, epidemiological investigation, and surveillance of citizens (38). In daily life, militaristic metaphors were used also in coverage of the existential threat to Israel's health system, especially by individuals within the system. Health services in Israel are provided mainly by a complex public health system that receives insufficient government funding. In recent decades the system has also had to contend, in a dynamic, competitive, and changing market, with an ever-stronger private health system. The lack of sufficient funding of the public health system and the attempts to revitalize it have generated a dynamic, turbulent, and threatening environment for health organizations and health system staff (2). During the pandemic period, media coverage and statements by members of the health system extended the narrative of the existential threat to the system by highlighting the distress of the hospitals and the public health system as part of the difficulty of coping with the pandemic. In this sense, the use of militaristic terms by clinicians helped create a sense of cohesion in the face of an external threat and merged with the media expressions of “winning the war” and the “battle against the virus” (36). However, this use of metaphors also served to generate feelings of stress and distress with the aim of pressuring decision makers to adopt policies that would benefit the health system (38).

The current study examines metaphoric linguistic expressions of clinical directors of COVID-19 wards that reflect thoughts, attitudes toward, and perceptions of their experience of directing a ward during the first wave (11, 15, 39).

Analysis of the conceptual metaphors in the directors' attempts to cope with a sudden epidemiological outbreak and an examination of their context at various levels may broaden the existing knowledge regarding metaphors and effective and ineffective coping among medical directors in crises. Therefore, our research questions are: (a) Did the COVID-19 ward directors use figurative language and metaphors during the crisis? (b) What kind of figurative language and metaphors did they use? How did they frame the situation? and (c) How did the use of figurative language shape their coping with situations of crisis and emotional overload?

## Methodology

This qualitative study focuses on an analysis of the discourse in in-depth interviews (20, 21, 40, 41), mapping the source and target metaphors (42) and their positioning in the coping process of ward directors (20, 41).

### The Study Population and Data Gathering

Both authors conducted in-depth interviews (45–60 minutes each) digitally via Zoom with 14 of the 21 directors of COVID-19 wards in Israeli hospitals during the pandemic's first wave (March–June, 2020). The data also included a corpus of 21 interviews with directors of COVID-19 wards, which were published in the media at the end of the first wave (June 2020). This corpus provided a reliability check and reinforcement of the findings of the analysis of the main body of data.

All the interviewees agreed to be interviewed and gave written permission for the interview to be recorded and transcribed and for parts of it to be published, on condition of anonymity. The participants were told that they had the right to end the interview at any point. Approval of the study was granted by the ethics committee of the institution with which the first author is affiliated.

### The Interviews

At the outset of the interview, the interviewer stated that the purpose of the study was to understand the experiences and thoughts of directors of COVID-19 wards. The interviewer asked one general question: Can you describe your experiences in heading a COVID-19 ward upon, and following, the outbreak of the COVID-19 pandemic? Most of the interviewees needed no clarification and told their stories without hesitation. They were encouraged to tell their stories in their own words, allowing a glimpse of their inner social world and of their experiences as an important repository of the meaning of the story told (43, 44).

The interviews were transcribed immediately in accordance with the rules of transcription. These rules require that the transcriber preserve the discourse precisely by writing down all the words heard in the interview, including hesitations, pauses in speech, and parts of words, and sounds that are expressions of feelings and thoughts—such as laughter, crying, stuttering, and sighing—that might reflect the inner state of the interviewee (43). This type of transcript is used mainly when the researcher is interested in investigating the “what” that is said and “how” it is said (45). The analysis of these interviews and the interviews in the press was conducted in an identical manner.

### Analysis of the Findings

The analysis was conducted in three stages. In the first stage, the researchers focused on identifying figurative linguistic means. The identification of a metaphor was conducted in accordance with Schmitt's model: identifying a word or phrase that can be understood beyond its literal meaning by understanding its context, identifying the source area that derives from a physical or cultural experience, and conceptualizing and assigning a source area to a target area (42). An expression is considered metaphorical when its contextual meaning is contrary to, different from, or broader than its literal meaning. Identifying metaphorical constructs that the interviewees generate and determining their source areas makes it possible to preserve their context and affinity to a linguistic construct as it appeared in the interview (18). At a later stage, every metaphorical expression was assigned to a broader concept.

In the second stage, the source areas were assigned to one of three levels—micro, meso, or macro—in accordance with their initial assignments. The micro level included an individual level (in accordance with the individual's characteristics and experience). The meso level was an organizational level (in accordance with the organization's behavior at the managerial, cultural, and financial levels). And the macro level was constructed from the environmental and socio-political context (the nationwide health system and all citizens), as is customary in the analysis of sociological theories.

The researchers resolved disagreements between them by discussing them. Mapping of the source areas and the target areas and assigning them to the micro, meso, and macro levels enabled the creation of a broad picture and an understanding of how the doctors used the source areas to conceptualize their coping with their COVID-19 experiences and their thinking about these processes on the three levels: personal, organizational, and social.

In the third stage, the researchers conducted an analysis of the interviewees in relation to their coping process. Accordingly, source areas were assigned to positive and negative thoughts and positive and negative coping (20, 21, 41). A source area indicating positive thinking was defined as desirable (assigning positive feeling to a new situation), whereas a source area indicating negative thinking was defined as undesirable (negative feeling following an unwelcome change). Positive coping was defined as the interviewees' being active (for example, taking initiative or generating an idea or a plan), whereas negative coping was defined as the interviewees' being activated by external forces over which they had no control—for example, bureaucracy, an unfamiliar virus, or the lack of a protocol (20, 21).

### Research Findings

The findings reveal widespread use of military expressions and metaphors, including military analogies and descriptions of military and wartime experiences in both sets of data—the interviews and the published corpus. Other conceptual metaphors or the use of examples from, or comparison to, other areas were relatively rare.

The common use of these metaphors in the media and in everyday discourse, including medical discourse, demonstrates the ever-present awareness of war, which reflects and creates a state of emotional distress and stress at every level.

We're at war against an unknown virus.

It has the feel of an 8 order [immediate mobilization of the reserves]; everyone is called up, everyone volunteers, everyone helps. The wind is a wind of war.

The source areas that were identified were assigned to several topics at the highest level. These topics included the hospital as the site of a battle, the nature of the crisis management, management of the ward, staff, teamwork, dangers and difficulties in contending with uncertainty, planning for the future, and personal topics such as feelings, the perception of cost, and the perception of reward. The source areas were divided in accordance with the micro and meso division—personal or organizational—and the macro division—environmental, political, social, in accordance with the target area and the context. Thus, for example, some areas found expression on two levels and some found expression on only one. This division was made on the basis of elements that have a primary, immediate effect rather than a secondary, long-term effect. Of course, elements that have individual effects and even broad organizational effects also have long-term effects in macro social and economic terms. Thus, for example, topics such as personal price and difficulties that have an immediate effect were assigned to the micro–meso area. However, some of these topics also have a secondary effect in the macro area. For example, such topics as personal sacrifice and difficulties that have an immediate effect at the meso-organizational level were assigned to that level, but some also have a secondary effect at the macro level. Thus, for example, the individual price has ramifications for aspects of cost, work burden, and teamwork, and these aspects have a broad effect not only on the organization itself but also on the entire system at the social and economic level.

All the source areas and target areas are presented in Table 1.

**Table 1:** Main source areas and target areas.

			**Micro**	**Meso**	**Macro**
**Definition of area**	**Source area**	**Target area**	**individual**	**organizational**	**socio-political**
Coping with COVID-19 like a war	The hospital as a field of war	Description of what is taking place in the hospital	√	√	√
	A battlefield		√	√	√
	A war front		√	√	√
	A struggle front		√	√	√
	The front is us		√	√	√
A war emergency situation	An emergency order	Feeling of immediate	√	√	√
	An illness that has burst into [our] lives	importance	√	√	√
	A wind of war	Sacrifice	√	√	√
	Immediate call-up [of reserves]	Threat	√	√	√
Managing the crisis	Military/mission-oriented management	Description of management		√	√
	Operations room	Daily description		√	√
	Orders	Organizational order		√	
	Evaluation of the situation	Organizational stability		√	√
	Daily evaluations of the situation			√	
	The hospital in military format			√	
	Army			√	
	Mossad			√	
	Policy evaluations				
Managing the workforce	Recruiting staff Volunteering for this task all hands on deck [sharing the burden]	Description of the process of building a staff array	√	√	
		Work force	√	√	√
		Sense of mission, meaning, friendship, empowerment, feeling of destiny, recognition of importance		√	
The nature of the job	The medical spearhead	Sense of mission	√	√	√
	Vanguard	Importance of the job		√	
	Leading the forces			√	√
The director's roles	The director as an officer		√	√	
	Personal example		√	√	
	The commander's resilience		√	√	
	The corona soldiers		√	√	
The staff's teamwork	The staff's comradeship	Friendship	√	√	
	The staff's cohesion	There is someone to rely on	√	√	
	On the same wavelength Auxiliary forces	Feeling of partnership	√	√	
	Not alone in the battle	Togetherness	√	√	
Cost and difficulties	Alone in the battle	Loneliness	√	√	
	Battle fatigue	Great	√	√	
	PTSD	difficulty	√	√	
	Battling for equipment	Contending with a crisis	√	√	
	Conditions and service	Work conditions	√	√	
Dangers	Running ahead	Paying a personal	√		
	Drawing fire	price	√		
	Exposed on the battlefield	Fear	√		
	Threat to [my] health	Personal sacrifice	√		
Feelings	An invisible enemy	Fear	√		
	Marching ahead in a fog of uncertainty	Anxiety	√		
		Helplessness			
		Uncertainty	√		
Reward	Heroism	Meaning	√	√	
	Salute	Respect	√	√	√
	Medal for heroism	Love	√	√	√
	The background behind the wings [a higher level of military decoration]		√	√	√
	Military decoration		√		
	Food shipments arrive		√		
	Esprit de corps		√		

The quotations highlight their context in the discourse analysis and the positive or negative interpretation given to them in the context and use of language. The division of the source areas into levels and their influence on framing and cognition were evident in the interviews. Below, we present findings by source areas assigned to all the levels and source areas associated with the micro-personal and meso-organizational level.

### Sources Assigned to All the Levels

Doctors identified several source areas as belonging to all the levels. These included broad areas of comparison to war, the analogy of the hospital to a battlefield, the definition of a military state of emergency, and the reward for their efforts, which had personal, organizational, and environmental contexts.

The days of the corona ward were days of grace. The spotlight was aimed at the front, as in every war, and the front is us. All the resources, all the equipment, all the empathy and sympathy—everything flows toward us.

The feeling was like that of receiving an immediate call-up order—you get a phone call, you drop everything and come to the meeting point without knowing any more details, but you understand that it's important.

Everyone is mobilized, everyone volunteers, everyone helps. Everyone was connected, as if it got some kind of prioritization at the national level, and everyone was there.

The use of these analogies enables a feeling of shared destiny, from the national level through the organization itself to the personal level. It is a feeling of belonging to something large, a destiny shared with the country's entire population. Yet, at the same time, there is a differentiation of the medical staff by their comparison to an elite unit, a path-breaking vanguard marching ahead of the troops.

Internal Medicine C [ward] was created—but outside this whole array, as a general-staff unit, a federal unit.

The shared work during this critical period created a shared destiny and a feeling of having a mission and of being the medical vanguard. This also gave the other people in the hospital peace of mind.

This comparison enabled the development of esprit de corps, a feeling of uniqueness and of a personal and organizational mission. The staff of the COVID-19 wards received the respect of others in the organization and were accorded priority in meeting the ward's needs. In place of the usual hierarchy based on level of education and position within the hospital, the feeling of the esprit de corps of an elite military unit was created.

Entry to the ward was managed like a mission and not necessarily according to rank. When a doctor entered the ward, sometimes he was also required to make beds and remove trash.

We also came to value all the “auxiliary forces” of the hospital that worked with great dedication, from the administrative head to the last of the maintenance staff who safeguarded the cleanliness of the department and our lives.

Together with a sense of pride, mission, and the importance of the role, the metaphors revealed feelings of personal sacrifice, self-endangerment, and fear.

There is uncertainty and pressure. Clearly there are concerns, and people are slightly afraid, but in the end, we understood that we're doing something very important that depends largely on the quality of our work.

You try to expose as few staff members as possible, then you go into the ward with a very small rescue force and work a lot harder.

Source areas connected to managing the ward and the staff also found expression at all levels—micro, meso, and macro. Thus, expressions such as “mobilizing the work force”, “volunteering for the mission,” and “all hands on deck” served to conceptualize the sense of mission, the heightened sense of shared destiny, and recognition of the importance of the role and mission at all three levels as part of the war effort.

I want to thank the ward's staff—medical, nursing, paramedical, and maintenance—and to honor them for mobilizing immediately and getting all hands on deck…despite the fear and panic that were in the air.

Everyone saw the total mobilization, the esprit de corps, and the solidarity of everyone who dealt with the sacred work in this war.

When I was asked to take on the mission of isolating and treating corona patients, it was clear to me that the ward's staff would step up to this mission with full force, precisely because of the sense of mission, of the task, and of marching ahead of the troops.

Source metaphors in the area of management of military operations, such as “operations room,” “combat theory commands,” and “daily evaluations of the situation,” also enabled organizational order, security, and stability at the micro, meso, and macro levels, in parallel to the national level at which the army and other military institutions were helping to manage nonmedical aspects of the crisis.

The hospital's behavior became the behavior of a military unit. We had an evaluation of the situation every day, there was a group of commands…It was very, very helpful.

The hospital prepared itself in a military-like format: daily evaluations of the situation, an operations room, everything was analyzed.

Another source area expressed at all levels was the reward for the actions of the medical staff: recognition of heroism, decorations for heroism, war decorations, and even deliveries of food, generating a sense of pride, gratitude, and respect.

Look at the staff members who continue their devoted work and salute them. They're true heroes.

The places that treated corona received the background behind the wings [a higher level of military decoration], the war decoration.

All the resources, the equipment, the sympathy, and the admiration—it all flows toward us…Every afternoon they also [deliver] the best of Israeli cuisine to pamper the corona soldiers. A euphoric mood, a sudden gentleness in the internal medicine ward, suddenly...the medal of valor [is] on [one's] chest!

### Source Areas Associated With the Micro-Personal Level and Meso–Organizational Level

A number of source areas are prompted by, and reflect, micro-meso levels only. They include personal and managerial aspects, some of which are related to the nature of the work and therefore are less relevant to the macro context. An example is the staff's work. The staff's cohesion is a crucial element in building trust, esprit de corps, and shared destiny, like that in the elite military units in which cohesion is crucial on the battlefield.

The staff who entered the ward in protective dress knew that they were not alone in the battle. Their colleagues in the team backed them up and watched over them…constantly from the control room, directed and guided their actions. The feeling of solidarity and mutual responsibility increased the trust between the members of the staff.

When I look in the eyes of the staff members with whom I was on the battlefield…I see a person who is on the same wavelength as me and we understand each other without words.

The feeling of watching out for each other and support by the staff appeared in several metaphors, including the naming of the administrative and paramedical staff members as “auxiliary forces”: “All kinds of auxiliary forces including teams that are paramedics”; “Auxiliary forces” of the hospital's administrative teams and others worked with great devotion'.

Source areas that were relevant only to the micro–personal level—such as the price individuals had to pay—were prominent. Expressions such as “running forward in battle,” “exposed on the battlefield,” “alone in the battle,” “battle fatigue,” and “post-traumatic stress disorder [PTSD]” shed light on phenomena such as the interviewees' fear, anxiety, and loneliness in battle.

We all experienced it…when you run ahead into fire,...you're the first one who draws it.

I'll give you an analogy…It's like battle fatigue.

A short time before that I saw a series about Chernobyl, and the first few times I felt as though I were in a similar situation facing an invisible enemy. There are no physical feelings of danger, but you are very tense in the wake of reports about the morbidity and mortality of doctors in Italy.

We learned to march ahead into battle from the battle fog of uncertainty, to rely on intuition, on gut feelings.

The analysis revealed great use of metaphors and examples based on personal experience from the time the doctors were soldiers and officers in the army and in the wars.

About 200 years ago I was…a crew member on a submarine. So, often …I said to them, “Look, it”s very similar'.

I remember it also from other wars, from the First Lebanon War and things like that.

Most of the people here have been in the army. I was in the army for six years. Here that counts as if I'm a total civilian.

The personal war experience shaped the form of management and how the event was perceived also at the experiential level, such as familiar feelings of anxiety, fear, and stress following the outbreak of war. “The corona disease burst into our lives like a storm.” “The intensity of the anxiety that I felt for my family, for everyone, is quite similar to the first night of the Gulf War”.

At the same time, the wartime experience made it possible to rely on the familiar and the known, which provided a sense of order and organization amid the chaos.

In Israel, we are militaristic by nature. We all were [in the army], if not sergeants or lieutenant colonels. But yes, the drill is like in a military operation... [Maybe] that was what did the job.

The use of personal military experience is also a by-product of the socio-cultural context in a country in which most of the citizens are former soldiers. To a great extent, even the running of the country is defined by military experience.

All the behavior was military behavior. All the operations room and the terms, and the army that entered the Ministry of Health, and the Mossad that sat inside the Ministry of Health. And the evaluations of the situation…[all] military concept[s].

The interviewees compared the tasks of managing the ward to the tasks of officers in a war, even though a ward director is normally concerned mainly with medical management. This comparison of the ward director to a commander of an elite unit on the battlefield is not trivial, and it is evidence of the need to enthuse people, to set an example, and to contend with the staff's fear and anxiety.

The directors were there all the time. The directors went inside; that is, the staff did not feel that they were being sent into the battle without the director shouting “After me!”

The director has to display this strength. An officer in the army also does this kind of thing.

It doesn't matter whether you're managing a corona epidemic in a hospital or some elite unit in the army.

The levels of authority and leadership were higher than the levels of management...In the everyday I'm often just a manager.

These metaphors demonstrate the difficulty of ward directors in a health system with a shortage of job slots, resources, and funds, crowded internal medicine wards, and concern for the daily functioning following the crisis.

The wonderful but starved health system must be revived, equipped, and strengthened so that it can continue to carry out its professional tasks reliably... both in times of peace and in times of war and epidemics.

Our elasticity is enormous. We proved it in the lightning war of the coronavirus and we are proving it day by day in the unbearably long war of attrition.

These statements exist alongside the expression of the personal struggle of returning to everyday difficulties.

We would receive…donations and meals…and attention in the media. Suddenly everything disappeared. You go back to being just another cog in the system and perhaps people now already want to forget you...and that is very, very difficult for some of the people.

Assigning source areas and target areas to the various levels in the world of the interviewees provided a glimpse of their coping mechanisms while demonstrating the difficulties and feelings—the price and the reward, as they saw it—of the state of distress they encountered while trying to contend with and manage the crisis in its beginnings. “And thus, we created the paths of action, on the fly”.

Framing the event as a battle, based on past experience and on known terminology while building esprit de corps and cohesion of the staff, enabled coping and construction of a narrative of a mission. “In the end it's how you build esprit de corps, how you build a narrative, how you build the feeling of excellence, of a mission”.

### Mapping the Interviewees' Modes of Coping and Thought

In the analysis, we mapped metaphorical constructs that were evidence of positive coping and thought and others that were evidence of negative and ineffective thought. Positive coping and thought were defined as the points at which the interviewees positioned themselves as active in the process, initiating, responsible, and instigating, and declared that the process was desirable. The following are examples.

We're under emergency orders; everyone pitches in to [accomplish] the task.

A euphoric feeling; suddenly the internal medicine ward is prestigious again.

Ineffective coping, on the other hand, was defined as a process in which the interviewees positioned themselves as passive and activated by forces over which they had no control, such as the pandemic and bureaucracy; experienced stress and tension; and declared the situation as undesirable.

It's like in war; you want it to be over in a day or two, but it doesn't end.

[It's]like in the Gulf War and the intensity of the anxiety I felt for my family, like the first night that the missiles were flying.

The source areas and their mapping are presented in Table 2.

**Table 2:** Source areas and modes of positive and negative coping.

**Positive/Negative**	**Source areas**	**Micro personal**	**Meso organizational**	**Macro socio-political**
Use of metaphorical constructs in positive coping	Mobilization and volunteering	√	√	√
	Military management	√	√	
	Auxiliary forces	√	√	
	Vanguard	√	√	
	Elite unit	√	√	
	Personal example	√	√	
	Heroism		√	
Use of metaphorical constructs in positive thinking	Esprit de corps	√	√	√
	Salute	√	√	√
	Pilot's wings	√	√	√
	Medal of valor	√	√	√
Use of metaphorical constructs in negative coping	Invisible enemy	√		
	Marching in the fog of battle	√		
	Fog of uncertainty	√		
	Cost of war	√		
	Exposed on the battlefield	√		
	The first who draws fire	√		
	Battle fatigue	√		
	Battling for equipment	√		
Use of metaphorical constructs in negative thinking	PTSD	√		
	Like an atom bomb	√		

## Discussion

This study analyzed metaphors and their role in framing reality and shaping coping mechanisms among COVID-19 ward directors in Israel during the first wave of the pandemic there. The study makes a number of contributions. Theoretically, it broadens the existing knowledge regarding military metaphors in the discourse between doctors and patients to include metaphors used by doctors in a health crisis. Methodologically, the study analyses the discourse in three stages: identifying source and target areas; assigning metaphors to macro, meso, and micro levels; and mapping the interviewees' statements by examining the context and ramifications of effective coping with stressful events. On the practical level, we propose interventions for building effective coping mechanisms for doctors and medical staff in healthcare organizations in times of crisis.

### The Use of Metaphors; Source and Target Areas

Doctors who headed COVID-19 wards made extensive use of metaphorical expressions and analogies from the military domain. The military source areas illustrate and mediate the interviewees' cognitive state of war and thus reflected and drove the cognitive framing of the situation. This framing enabled the creation of mechanisms for coping with the sudden distress by drawing on personal and collective experience (8, 9, 12, 13, 17). Thus, for example, the source areas that presented coping with COVID-19 as being like a war included “the hospital as a battlefield”. The mediation of the feelings of urgency, emergency, and immediate distress led to the use of metaphors that reflect a state of emergency, such as “emergency order,” which generated a feeling of immediate importance, threat, and stress. Framing this situation led to the immediate need to manage the crisis and to the use of military metaphors from this source, such as “military/mission-oriented management” and “operations roo.” Such framing enabled the directors to mediate target areas of management and organization that create stability and organizational order.

The directors also used these source areas to mediate managerial roles that were compared to roles of military command—for example, “personal example of the leader.” They also expressed the need to manage and consolidate the work force in the wards: “mobilizing a work force” and “fighters' esprit de corps”. This framing made it possible to build stability and order while generating motivation and a sense of mission that differentiated the COVID-19 staff as a “medical vanguard” or “elite unit”. This was backed up by the great reward, also conceptualized in military terms, such as “war decoration,” which also found collective expression, as in the food deliveries and admiration, and connected with the feelings of meaning, respect, and love.

Conceptualizing values that are basic on the battlefield, such as comradeship, cohesion of the unit, and “combatants' esprit de corps,” contributed to the feeling of stability, cohesion, and shared destiny of the medical team while drawing on their individual and collective experience. The doctors also presented metaphors from source areas that conceptualize dangers and difficulties, such as “alone in the battle” and “battle fatigue”, which reflected and framed distress and difficulty, a high personal price, personal sacrifice, and fear, loneliness, and anxiety.

### Framing the Reality in Assigning Micro, Meso, and Macro Levels

The use of metaphors mediated and framed the experience while creating meaning and logic (7). Assigning the source areas to three levels makes it possible to examine the framing and creation of the cognitive reality as the interviewees saw it. The micro level is composed of the personal level, which includes the individuals and their characteristics, personal experiences, and immediate environment. The meso (organizational) level includes the organization in which they work and their organizational culture. The macro level includes the socio-political complex of the state. This level is greatly affected by processes and perceptions at the individual and organizational levels. In the long term, framing reality and perceptions at the individual and organizational levels will also indirectly affect the macro level—the environment and the decision makers in it.

In Israel, from the start, the pandemic was described in military terms, including “war” and “nationwide mobilization” (46, 47). The use of bellicose metaphors by journalists and politicians became a tool for enlisting civilian cooperation in many countries (48). Similarly, in Israel, positioning doctors as combatants in the vanguard of the struggle enabled the use of concepts of the heroism of the vanguard in an elite unit. This, in turn, helped create a narrative of having a mission, importance, and personal and organizational meaning, accompanied by admiration of the public and the media. This framing of the situation created a sense of comradeship and individual and collective empowerment, which enabled acceptance of the price of working in COVID-19 wards. However, excessive use of the narrative of heroism should be avoided, because it could have a negative psychological effect on health system staff and might make it difficult for them to define the limits of their obligation to provide medical treatment (49).

The source areas that created a feeling of having a mission, motivation, order, and organization of the work force took on a broader application on all levels. They intensified the feeling of emergency, shared destiny, the need to act immediately, mobilizing a work force, and managing it, and resulted in expressions of great admiration for the COVID-19 staff. But assigning the source areas that mediated experiences and feelings of fear and of personal cost only to the personal level emphasized the sense of isolation, which was evident also in the distancing from family members and colleagues and thus intensified the feeling of being alone in the battle. This was true also of feelings of fear, anxiety, and isolation as part of the price they paid.

These findings support previous studies that viewed the use of metaphors as a double process (14, 15) and their use to construct the situation as motivated by contexts at the macro and micro levels and influenced by personality, life experience, and cultural characteristics (12, 13).

### Effective and Ineffective Coping Mechanisms

The use of metaphors that had a positive impact enabled effective coping with a complex reality. Metaphors that had negative contexts constituted ineffective mechanisms of coping. The war metaphors that promoted effective coping created a sense of order and coherence, like the reality-construction and sensemaking processes in understanding the environment through order and logic based on past experience (50). Managing in accordance with orderly rules and a clear daily routine enabled a reframing of the chaotic reality according to familiar patterns (51).

In contrast, military metaphors such as “battle fog,” and “loneliness in battle,” reflected, generated, and intensified feelings of isolation and loneliness, helplessness, uncertainty, and sacrifice, and contributed to intense feelings of being stuck and fear among some interviewees. Just as doctors' use of military metaphors that are linked to traumatic experiences of difficulty and isolation on the battlefield may generate antagonism in some patients, so such metaphors may arouse in the staff a framing of the situation that matches those experiences (5, 47). The extensive use of military metaphors may generate panic, paternalism, reduced effectiveness of coping, and resistance to cooperation among the public, and in the case of COVID-19 might lead to increased hospitalization of patients (5, 23). The ramifications of the use of military metaphors for framing a situation vary from person to person (7, 23). Also, the ramifications of the use of these metaphors by the medical staff may have a deleterious effect on the doctor, depending on the doctor's personality. Therefore, although the use of such language can be a powerful tool, its limitations and its effects on environmental and personality factors should be kept in mind. Allowance should be made for alternative metaphors so that an optimal fit can be found for the individual and the environment (23).

**Figure 1:**
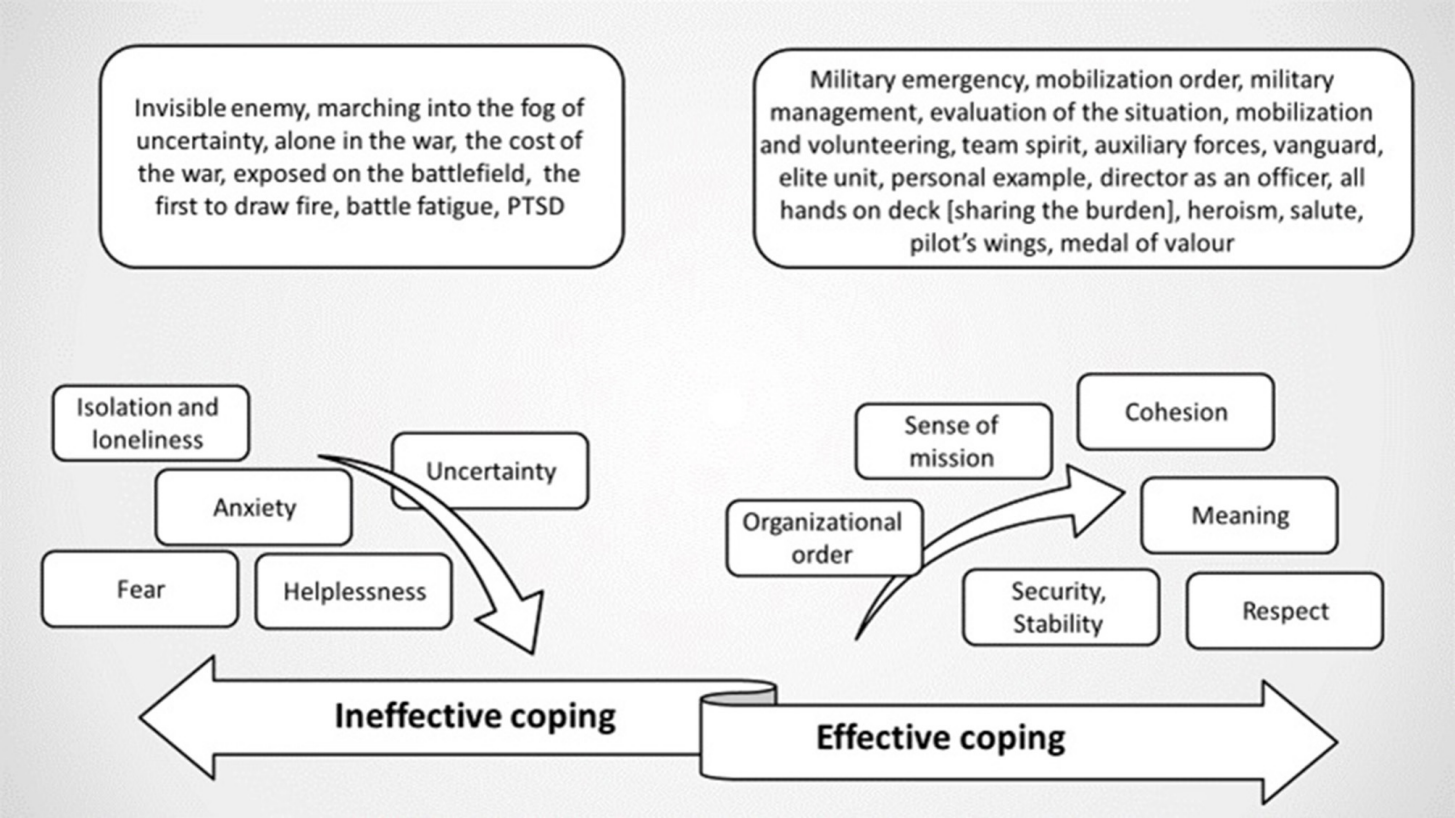
Effective and ineffective coping mechanisms.

Figure 1 shows the influence of the use of military metaphors and images on the contexts at the various levels.

## Conclusions and Recommendations

At the macro level, in Israel, as in other countries, the COVID-19 pandemic has been defined in militaristic terms in the political, public, and media discourse (46, 47). This broad use of such terms, as part of a siege mentality (36), has served as a means of maneuvering individuals toward cohesion and shared effort. The siege mentality is characteristic of Israel, whose population is experienced in drawing together against an external enemy. The use of such metaphors at various levels, cultural and cognitive, is characteristic of the Israeli mindset and narrative: The entire system is oriented, both organizationally (economically, industrially, and constitutionally) and mentally, toward constant preparation for war, as if this were the natural state of the world (32). Therefore, the use of such language in the general discourse, together with the use of such expressions in describing the health system in its entirety and the coping of the medical teams in particular, reinforced this mentality and made it possible to frame the situation as a battle against a common enemy. This framing helped reduce public criticism of the failures in managing and financing the crisis (46). It also achieved the public's cooperation in such health matters as quarantines and epidemiological monitoring (36, 52). The health system, from a sense of emergency, made media and political use of this militarism to float and justify previous broad demands, such as additional staff positions and funding for the health system, and justified and intensified criticism of the state policy of underfunding the health system.

At the micro–individual and meso–organizational levels, the use of this militarism relied on the individual experience of the members of the medical teams as part of the process of effective and ineffective individual and organizational coping. Undoubtedly, these processes preserved militarism at all levels in Israel and in this case made possible systemic coping with the COVID-19 crisis. However, these processes at the political level may lead to silencing of criticism of managerial and political behavior, which is an important aspect of democracy and making the system more efficient. At the personal and organizational levels, too, the use of metaphoric discourse and the inculcation of such values and principles as part of the discourse culture and of the organization is important, with the proviso that there be recognition of the limitations of this tool, which differ from person to person and are also influenced by the organizational context (23). Therefore, the use of such discourse must be accompanied by organizational, managerial, and even economic changes, such as emotional and financial support and concern, both at the organizational level and at the level of decision makers. These changes will strengthen the system and will reflect these empowering and strengthening values and perceptions under everyday conditions and in times of crisis.

To generate more effective coping among medical personnel and organizations in coping with crises, we propose using metaphors, analogies, and words that emphasize ideology and values that empower (heroism, cohesion, comradeship). We also propose avoiding metaphors, analogies, and words that emphasize distress and isolation (53). Sharing beliefs and values through selected metaphors, adapted to the individual in a given cultural context, may contribute to effective coping of doctors in a crisis by reframing the situation. Adopting metaphors that emphasize the meaning of the role and the importance of individuals and their actions—in addition to appreciation for their contribution to the organization—is essential for effective coping that motivates the individual and increases willingness to pay the personal, familial, and societal price. Integrating these values-laden expressions in the organizational discourse as part of the array of coping patterns may make it easier for individuals to reframe a complex reality.

Inculcating values and regulations that generate stability, such as leadership and planning, together with crisis-oriented leadership training of staff, can shape the perception of stability even in a crisis. Using these values as anchors in everyday situations may help increase the emotional wellbeing of ward directors and staff while fostering optimal functioning of the individual and the organization.

Using metaphors in the organizational discourse may reduce the staff's feelings of isolation and personal sacrifice and may contribute to the construction of an array of supportive defense mechanisms for everyday conditions and crises. It may also prevent destabilization and less effective coping in crisis situations. The contribution of military metaphors that emphasize staff cohesion, training, comradeship, and partnership cannot be overstated. Using metaphorical discourse and funding activities aimed at generating cohesion through such discourse are crucial for clinicians' wellbeing and for their optimal functioning.

## Data Availability Statement

The original contributions presented in the study are included in the article/supplementary material, further inquiries can be directed to the corresponding author/s.

## Ethics Statement

The studies involving human participants were reviewed and approved by Hadassah Academic College. The patients/participants provided their written informed consent to participate in this study.

## Author Contributions

LN-S: conceptualization, data curation, methodology, writing-original draft preparation, writing-reviewing, and editing. GG: conceptualization, data curation, writing-original draft preparation, writing-reviewing, and editing. All authors read and approved the final manuscript.

## Conflict of Interest

The authors declare that the research was conducted in the absence of any commercial or financial relationships that could be construed as a potential conflict of interest.

## Publisher's Note

All claims expressed in this article are solely those of the authors and do not necessarily represent those of their affiliated organizations, or those of the publisher, the editors and the reviewers. Any product that may be evaluated in this article, or claim that may be made by its manufacturer, is not guaranteed or endorsed by the publisher.
